# Antibiofilm Properties of Temporin-L on *Pseudomonas fluorescens* in Static and In-Flow Conditions

**DOI:** 10.3390/ijms21228526

**Published:** 2020-11-12

**Authors:** Angela Di Somma, Federica Recupido, Arianna Cirillo, Alessia Romano, Alessandra Romanelli, Sergio Caserta, Stefano Guido, Angela Duilio

**Affiliations:** 1 Department of Chemical Science, University of Naples “Federico II”, Via Cinthia, 80126 Naples, Italy; angela.disomma@unina.it; 2 Division of Chemical Technology, School of Chemistry, Aristotle University of Thessaloniki, University Box 116, 54 124 Thessaloniki, Greece; federecu@chem.auth.gr; 3 Department of Chemical, Materials and Industrial Production Engineering, University of Naples “Federico II”, Piazzale V. Tecchio 80, 80125 Naples, Italy; sergio.caserta@unina.it (S.C.); steguido@unina.it (S.G.); 4 CEINGE—Biotecnologie Avanzate, 80145 Naples, Italy; cirilloa@ceinge.unina.it (A.C.); romanoa@ceinge.unina.it (A.R.); 5 Department of Pharmaceutical Sciences, University of Milan, Via Venezian 21, 20133 Milan, Italy; alessandra.romanelli@unimi.it

**Keywords:** biofilm, antimicrobial peptide, biofilm formation inhibition, dynamic and static condition

## Abstract

Biofilms consist of a complex microbial community adhering to biotic or abiotic surfaces and enclosed within a protein/polysaccharide self-produced matrix. The formation of this structure represents the most important adaptive mechanism that leads to antibacterial resistance, and therefore, closely connected to pathogenicity. Antimicrobial peptides (AMPs) could represent attractive candidates for the design of new antibiotics because of their specific characteristics. AMPs show a broad activity spectrum, a relative selectivity towards their targets (microbial membranes), the ability to act on both proliferative and quiescent cells, a rapid mechanism of action, and above all, a low propensity for developing resistance. This article investigates the effect at subMIC concentrations of Temporin-L (TL) on biofilm formation in *Pseudomonas fluorescens* (*P. fluorescens*) both in static and dynamic conditions, showing that TL displays antibiofilm properties. Biofilm formation in static conditions was analyzed by the Crystal Violet assay. Investigation of biofilms in dynamic conditions was performed in a commercial microfluidic device consisting of a microflow chamber to simulate real flow conditions in the human body. Biofilm morphology was examined using Confocal Laser Scanning Microscopy and quantified via image analysis. The investigation of TL effects on *P. fluorescens* showed that when subMIC concentrations of this peptide were added during bacterial growth, TL exerted antibiofilm activity, impairing biofilm formation both in static and dynamic conditions. Moreover, TL also affects mature biofilm as confocal microscopy analyses showed that a large portion of preformed biofilm architecture was clearly perturbed by the peptide addition with a significative decrease of all the biofilm surface properties and the overall biomass. Finally, in these conditions, TL did not affect bacterial cells as the live/dead cell ratio remained unchanged without any increase in damaged cells, confirming an actual antibiofilm activity of the peptide.

## 1. Introduction

Biofilm consists of a complex self-produced matrix of polysaccharides, DNA, lipids, and proteins that protect bacteria from the environment, including the host immune system [1]. Biofilms are ubiquitous in nature, having the ability to adhere to virtually any surface, either biotic or abiotic, including medical devices, such as catheters or prosthesis, causing chronic infections difficult to eradicate.

Biofilms greatly contribute to developing antimicrobial resistance by different mechanisms. Nutrient and oxygen depletion occurring within the matrix induces microorganisms to adopt a stationary phase making bacterial cells less sensitive to growth-dependent antimicrobial factors. Moreover, biofilms are more resistant to hostile environmental conditions, the clearance operated by the host immune system, and the flowing action of body fluids as it occurs in catheters and other medical devices [2,3].

Studies aimed to unveil molecular and cellular mechanisms underlying biofilm formation are usually performed in static fluid conditions to standardize experimental procedures in the absence of environmental complexities. However, static fluid conditions are usually very far from reality because biofilms formation often occurs under fluid flow that significantly affects their formation, composition, and architecture [4]. A classic scenario of biofilm formation under flow might be the ship hulls, which are generally prone to strong colonization of biofilms. Analogously, in the biomedical field, medical devices like catheters, prostheses, and heart valves exposed to organic fluids flow are often attacked by biofilm originating severe infections [5].

Therefore, a fundamental parameter that affects biofilm formation is the shear flow. During the attachment phase, the fluid flow directly affects the microbial interaction with surfaces, due to the presence of gradients in the fluid velocity. The shear stress, always generated near a solid surface, also affects the direction of cells swimming as recently demonstrated for *Bacillus subtilis* and *Pseudomonas aeruginosa* [6]. During the maturation phase, the shear might induce complex morphologies, such as microcolonies, tower-like, and filamentous structures [7]. Furthermore, shear flow promotes the production of the extra-polymeric matrix, alteration of bacterial gene-expression [8], a variation of the matrix viscoelastic properties, and regulation of the bacterial quorum sensing [9].

Different flow conditions are known to also affect biofilm morphologies. *Desulfovibrio* spp. (an anaerobic sulfate-reducing bacteria) and *P. aeruginosa* biofilms grown at higher shear were found to be smoother and denser, but also more rigid and stronger than those grown at lower shear rate conditions [10]. Moreover, biofilms formed under higher shear stress (i.e., in the turbulent regime) show a higher production of polymeric substances as reported by Brading et al. and Pereira et al. [11,12]. It is still unclear whether the increased density and strength of biofilms exposed to higher shear stress conditions are regulated at the genetic level or are determined by purely physical mechanisms, but it appears that exopolysaccharides (EPS) are fundamental for both the structure and cohesive strength of biofilms [13]. The shear flow might also affect bacterial susceptibility to antibiotics, since molecular diffusion promotes an antibiotic gradient within the matrix as reported by Salek et al. [14].

The microfluidic technique has been attracted to study biofilm formation under well-controlled flow conditions in the real-life mimicking the environment by establishing stable chemical gradients in highly throughout-manner [15]. The microfluidic technique requires really small operative volumes with a large surface/volume ratio, which enhances mass transport within biological systems [16]. In biofilm research, microfluidics has been used to investigate the role of hydrodynamics in local microenvironments [17], since it is possible to tailor the hydrodynamic conditions with the aim to achieve specific shear flow conditions, by also providing non-invasive imaging of biofilm architectures.

This new approach opens up the possibility to investigate the effect of the AntiMicrobial Peptides (AMPs) on biofilm development under flow conditions, with the aim to develop new strategies to inhibit and contrast biofilm formation in real biomedical applications. AMPs are short peptides produced by various organisms, which inhibit the growth or impair the survival of many bacterial species. AMPs are active against a broad-spectrum of microorganisms, including viruses, parasites, and both Gram-positive and Gram-negative pathogenic bacteria [18]. Additionally, many AMPs also show anti-biofilm activity against multidrug-resistant bacteria acting at different stages of biofilm formation, on disparate molecular targets, and with various mechanisms. (CSA)-13 can quickly penetrate into mature biofilms formed by *P. aeruginosa* and permeabilize bacterial cell membranes [19], whereas human cathelicidin LL-37 and indolicidin peptides might prevent biofilm formation of *P. aeruginosa.* Other AMPs are able to alter and reduce the architecture of an biofilm matrix by targeting polysaccharide intracellular adesine (PIA) or impairing EPS production [20].

The frog-skin-derived Temporin L (TL) has been widely examined in the literature, especially for its antibacterial and antibiofilm properties [21,22]. Recently, TL was shown to impair *E. coli* growth by inhibiting cell division, specifically targeting FtsZ, a protein involved in Z-ring formation [23]. However, the antibiofilm properties of TL have not been studied under flow conditions, which represent real cases in biomedical applications.

Here we report the investigation of the antibiofilm effects of TL on *Pseudomonas fluorescens* biofilm under both static and dynamic conditions. First, the antibiofilm properties of TL at different peptides concentration were tested under static conditions. Next, biofilms were grown under well-controlled flow conditions using a commercial microfluidic device to simulate real flow conditions in the human body. Preliminary in-flow tests were performed to define the effects of TL at different incubation times. Morphological quantification of the architecture of TL treated biofilms by fluorescent analyses demonstrated that TL displays antibiofilm properties.

## 2. Results

### 2.1. The Effect of Temporin L on P. fluorescens Cell Growth

Preliminary experiments were carried out on *P. fluorescens* to evaluate the antimicrobial activity of TL. Bacteria cells were grown in the presence of serial dilution of TL from 512 μM to 0.5 μM, and the MIC and MBC values were evaluated. Both MIC and MBC resulted in being higher than 512 μM. Moreover, the growth profiles in the presence of different concentrations of TL, confirmed that the AMP did not show significant effects on this strain (Supplementary Figure S1). All experiments on *P. fluorescens* biofilm were then carried out at subMIC concentrations of TL, i.e., 25 and 100 μM.

### 2.2. The Effect of Temporin-L on Biofilm Formation in Static Conditions

**Figure 1:**
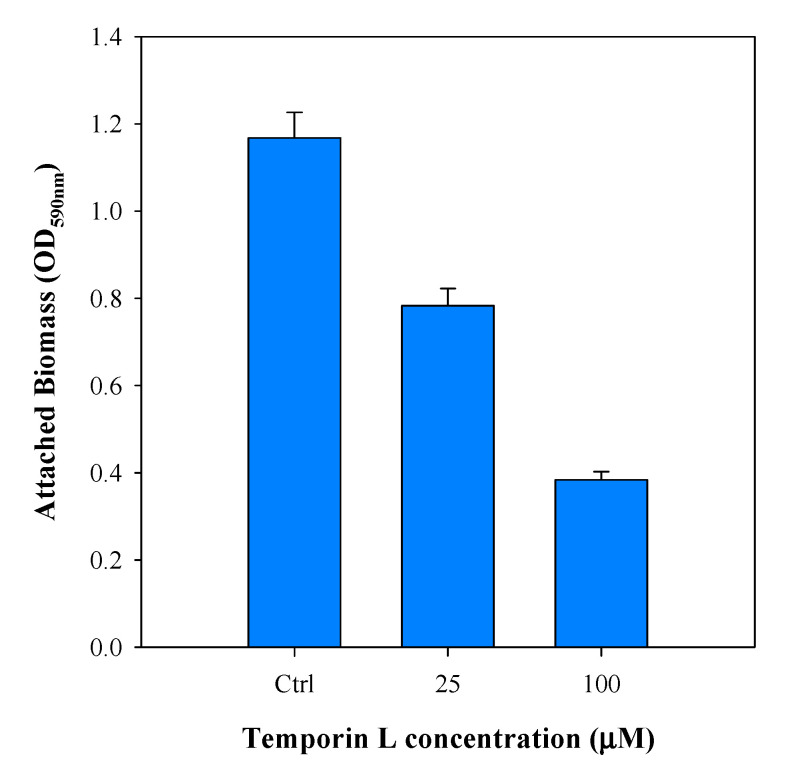
Estimation of attached biomass of 3 days-old-biofilms cultivated under stagnant fluid conditions using 12 well microplates (no glass substrates) upon TL treatment at two different concentrations (25 and 100 μM). Data were calculated from three independent measurements and are reported as average; the standard deviations are indicated.

The in vitro effect of TL on *P. fluorescens* biofilm formation was first evaluated in static conditions using 12 well-multiplates (without glass coverslips). Bacterial cells were inoculated in the presence and in the absence of two different concentrations of TL, 25 and 100 μM, respectively. Quantification of biofilm formation was monitored by the Crystal Violet assay. Figure 1 shows that TL decreased biofilm development in a dose-dependent manner displaying a reduction of about 75% in biofilm growth at 100 μM TL.

**Figure 2:**
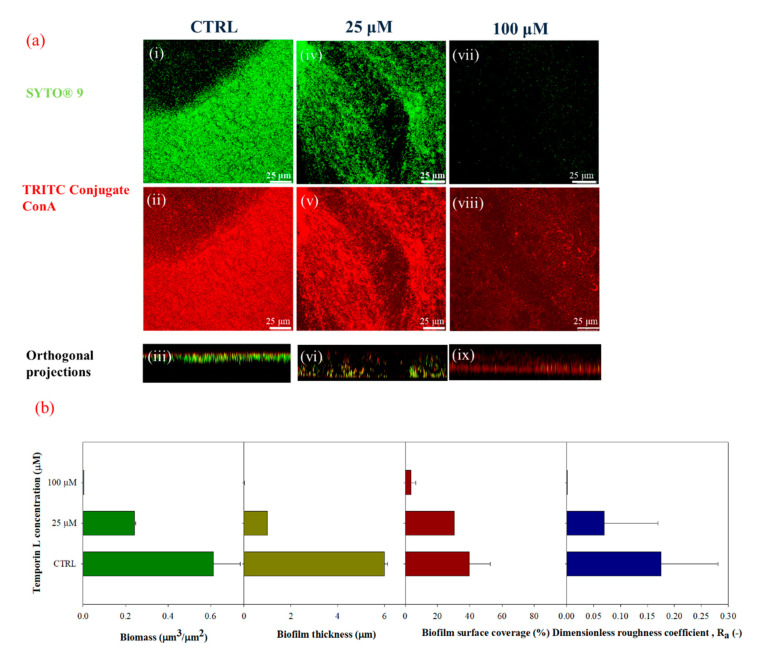
(**a**) CLSM images of 3 days-old-biofilms cultivated under stagnant fluid conditions in green (i, iv, and vii, SYTO^®^ 9, 3 μL/mL), red channels (ii, v, and viii, TRITC ConA, 100 μg/mL) and orthogonal projections (iii, vi, and ix) upon TL treatment at two different concentrations (25 μM and 100 μM). (i–iii) CTRL (negative control), (iv–vi) 25 μM, (vii–ix) 100 μM; 63X oil immersion magnification, scale bar = 25 μM. (**b**) Morphological parameters were evaluated by processing CLSM stack images of biofilms, cultivated under stagnant fluid conditions at different TL concentrations: Ctrl (negative control), 25 μM and 100 μM. Data were calculated from three independent measurements and are reported as average; the standard deviations are indicated.

The morphology of *P. fluorescens* biofilm formed on glass substrates was assessed by Confocal Laser Scanning Microscopy (CLSM) using a double-staining procedure. Alive cells were stained with the green fluorescent dye SYTO^®^ 9, while the red fluorescent dye Tetra-methyl-rhodamine (TRITC) conjugated Concanavalin A was used to stain α-mannose residues within the biofilm matrix. Figure 2a shows the CLSM images of TL treated and untreated biofilms cultivated under stagnant fluid conditions on glass substrates. Untreated bacteria exhibited a compact and thick biofilm matrix with complex architecture (Figure 2(ii), red fluorescence) in the presence of alive bacterial cells (Figure 2(i), green fluorescence), which appeared as agglomerates. At increased TL concentration, a clear alteration in the biofilm architecture was detected (Figure 2(v–viii)). Biofilm thickness significantly decreased upon treatment with 25 μM TL (Figure 2(v)) (Figure 2(iv)). Higher TL concentration (100 μM) led to a clear reduction of cell density (Figure 2(vii)) and to fragmentation of the polysaccharide matrix illustrated by the dispersion of the ConA (Figure 2(viii)).

This aspect was also corroborated by the quantification of CSLM images (Figure 2b). The attached biomass decreased by 61% at 25 μM and by 99.6% at 100 μM TL, respectively, while biofilm thickness was reduced by more than 80% at 25 μM being nearly negligible at 100 μM. Biofilm surface coverage also decreased as the peptide concentration increased by achieving an average surface coverage of 3 ± 1% of the total available surface at the concentration of 100 μM. Similar behavior was observed for the dimensionless roughness coefficient. These results suggested that disruption of the biofilm architecture had to be ascribed to a specific antibiofilm effect of TL. Moreover, MBEC value was calculated for TL, resulting around 256 μM.

### 2.3. The Effect of Temporin-L on Biofilm Formation under In-Flow Conditions

**Figure 3:**
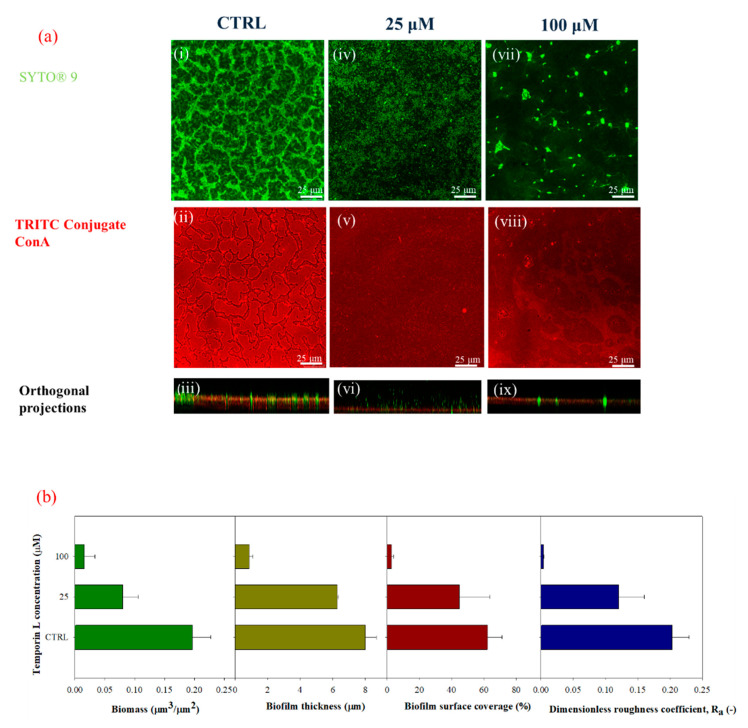
(**a**) CLSM images of 3 days-old-biofilms cultivated under in flow conditions in green (i, iv, and vii, SYTO^®^ 9staining, 3 μL/mL), red channels (ii, v, and viii TRITC ConA, 100 μg/mL) and orthogonal projections (iii, vi, and ix) upon TL treatment at two different concentrations (25 μM and 100 μM). (i–iii) CTRL (negative control), (iv–vi) 25 μM, (vii–ix) 100 μM. Experiments were carried out by dissolving TL in the bacterial suspension at the inoculation; 63X oil immersion magnification, scale bar = 25 μm. (**b**) Morphological parameters were evaluated from CLSM stack images of untreated and TL-treated biofilms (25 and 100 μM). TL was injected together with the bacterial suspension in the microfluidic system during the inoculation phase. Data were calculated from three independent measurements and are reported as average; the standard deviations are indicated.

Next, the effect of TL was investigated on biofilms cultivated under in-flow conditions. Biofilms were formed on a polymeric coverslip using a microfluidic chamber at the imposed volumetric flow rate of 0.1 μL/min, corresponding to a wall shear rate of 1 s^−1^. A first experiment was designed by assessing the action of TL on biofilm growth. The peptide was dissolved in the bacterial suspension, and the resulting solutions used as inoculum. Alive cells and biofilm matrix were stained following the protocol described before, and the results were assessed by Confocal Laser Scanning Microscopy (CLSM). Figure 3a shows the CLSM images of untreated biofilms (i–iii) and biofilms treated with different TL concentrations, 25 μM (iv–vi) and 100 μM (vii–ix), respectively, in the green-red channels and orthogonal projections. The morphological quantification of the peptide treated biofilms is displayed in Figure 3b.

The untreated biofilms showed clumping like morphology in which bacterial aggregates were separated by interstitial voids within the extra-polymeric matrix. Panels vii, viii, and ix, in Figure 3a, show that at the highest examined concentrations of the peptide, the biofilm matrix was progressively reduced, and its architecture disrupted, confirming the results obtained for TL-treated biofilms cultivated in stagnant fluid conditions. It should be pointed out that at a subMIC concentration of TL (100 μM) in these conditions, the biofilm matrix was almost totally discontinued as indicated by the low amount of red fluorescence.

A quantitative analysis of the biofilm morphology from stack-images was performed, and the results are shown in Figure 3b. The effect of TL on biofilm growth could be clearly inferred also under in-flow conditions. The attached biomass was reduced by 60% and 92% at 25 μM and 100 μM TL, respectively. The biofilm thickness, surface coverage, and roughness coefficient also decreased at increasing TL concentration, but the peptide showed different effects. At 25 μM, biofilm features were only slightly affected by TL treatment as the surface parameters displayed a limited reduction. At 100 μM, TL exerted a significant effect on biofilm surface parameters as biofilm thickness was reduced by 89%, the surface area decreased by 95.7%, and the roughness coefficient by 98%.

**Figure 4:**
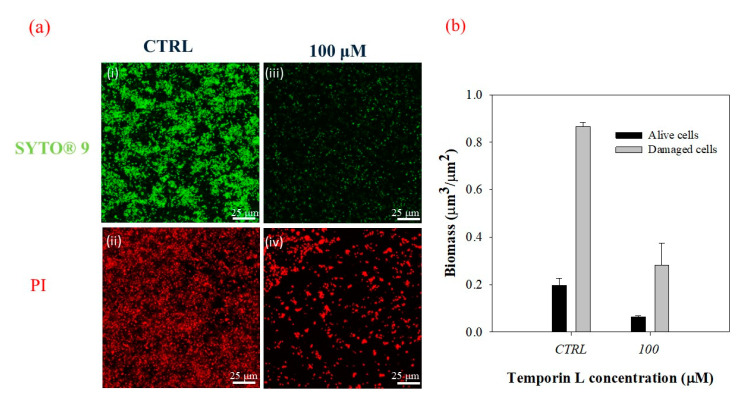
(**a**) CLSM images of 3 days-old biofilms cultivated under in flow conditions under the action of TL (100 μM). CTRL (i,ii), 100 μM TL (iii,iv). Experiments were carried out by dissolving TL in the bacterial suspension at the inoculation. Biofilms were double-stained with the LIVE/DEAD BacLight Bacterial Viability Kit. Alive cells were stained with the green fluorescent dye, SYTO^®^ 9 (3 μL/mL). Damaged cells were stained with Propidium Iodide (1 μL/mL). The staining procedure was performed at the same time; 63X magnification, scale bar = 25 μm. (**b**) Biomass (μm^3^/μm^2^) of alive and damaged cells in the function of the TL concentration. The alive and dead cells ratio was measured as 0.23 in the control, and 0.22 in the TL treated sample—confirming the antibiofilm activity of the peptide.

Then we confirmed that the disruption of the biofilm matrix had to be ascribed to an effective antibiofilm activity of TL using a double-staining method according to the LIVE/DEAD procedure. The morphology of *P. fluorescens* biofilm was examined using the experimental conditions previously described for the in-flow experiment where alive cells were stained with the green fluorescent dye SYTO^®^ 9 and dead cells with the red fluorescent dye Propidium Iodide (PI). Figure 4a shows the CLSM images acquired in the absence (Figure 4(i,ii)) and in the presence (Figure 4(iii,iv)) of the peptide at the subMIC concentration of 100 μM. Qualitative investigations of confocal microscopy analyses demonstrated that in these conditions, a substantial number of bacterial cells were still alive, as indicated by the amount of green fluorescence.

Figure 4b shows a quantitative evaluation of live and dead cells from the morphological analysis of z-stacks. It should be underlined that in these conditions, the number of dead cells were expected to be always higher than live cells, due to the length of the experiment and the flowing conditions. However, quantitative measurements demonstrated that TL did not affect bacterial cells as the live/dead cell ratio remained unchanged without any increase in damaged cells, confirming an actual antibiofilm effect of TL.

We also evaluated the ability of TL to affect mature biofilm by a series of experiments carried out in dynamic conditions by flowing the antimicrobial/nutrient medium solution on preformed biofilms. Following *P. fluorescens* attachment and biofilm development, microfluidic channels were supplemented with a growth medium containing 100 μM TL under flow conditions for a further 24 h. The control channel was treated in the same conditions except that TL was absent. Alive cells and the biofilm matrix were stained, as above described.

**Figure 5:**
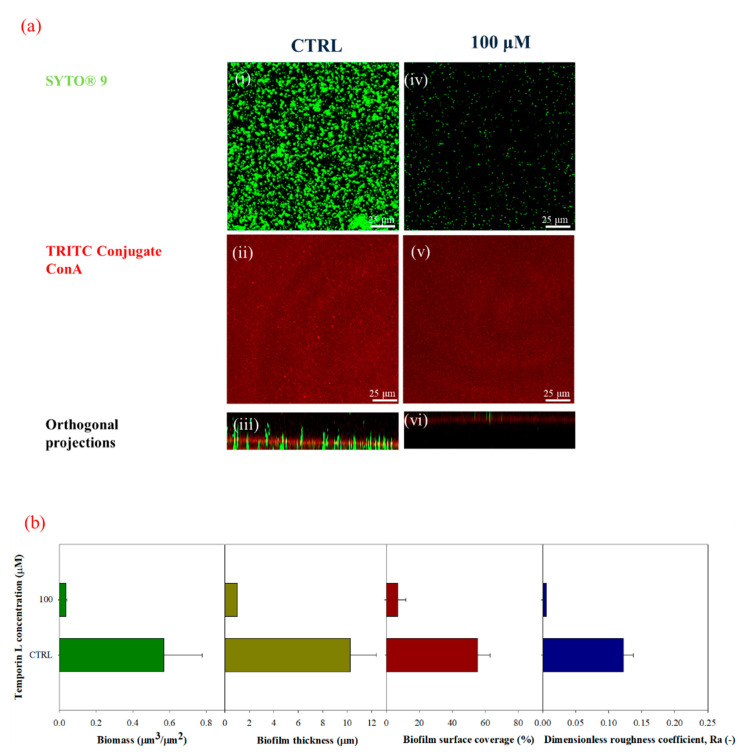
(**a**) CLSM images of biofilms cultivated under in flow conditions in green (i,iv, SYTO^®^ 9stain at the concentration of, 3 μL/mL) red channels (ii,v TRITC ConA staining, 100 μg/mL) and orthogonal projections (iii,vi) upon TL treatment (100 μM). TL effect was investigated on three days-old biofilms under flow for 24 h. (i–iii) CTRL (negative control), (iv–vi) 100 μM; 63X magnification, scale bar = 25 μm. (**b**) Morphological parameters were evaluated from CLSM stack images of untreated and TL-treated biofilms (100 μM). TL was dissolved in the nutrient medium solution, and the resulting solution was conveyed to the preformed biofilms (after 72 h of incubation) for 24 h. Data were calculated from three independent measurements and are reported as average; the standard deviations are indicated.

Figure 5a shows the confocal microscopy images acquired in both green and red channels in the presence of 100 μM TL (iv–v, and vi) compared to the negative control (i–iii). Figure 5(v) shows that following TL treatment, a large portion of the biofilm architecture was clearly perturbed (red fluorescence), and a number of live cells could still be observed (Figure 5(iv), green fluorescence). These data suggest that TL might penetrate the preformed biofilm architecture with a still unknown mechanism causing biofilm eradication. Quantitative measurements of biofilm morphology are reported in Figure 5b.

These results suggested that TL might also exert its antibiofilm activity on preformed biofilms as a reduction of 10-fold in biofilm biomass was detected. Similar behavior was observed for the maximum biofilm thickness, as well as for the surface area covered by biofilms that was reduced from 56% to 7%. The dimensionless roughness coefficient was close to zero, which led to argue that biofilm eradication might occur, since the red fluorescent signal of TRITC ConA was also very low, not allowing the evaluation of the roughness parameter through image analysis technique. The results obtained were pretty comparable with those obtained by TL treatment during bacterial growth and biofilm development supporting the idea that TL might be able to penetrate the biofilm structure leading to a substantial eradication of the preformed biofilm.

**Figure 6:**
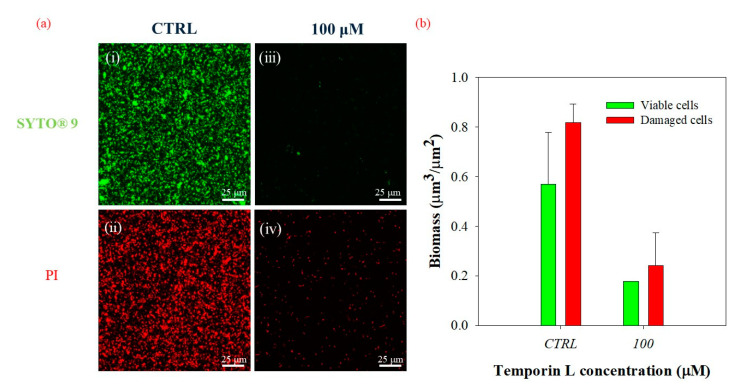
(**a**) CLSM images of biofilms cultivated under in-flow conditions. (i,ii) CTRL (negative control), (iii,iv) TL treated biofilms (iii,iv, 100 μM), which was dissolved in the nutrient medium and flowed for 24 h. Alive bacterial cells were stained with the green fluorescent dye, SYTO^®^ 9 (3 μL/mL), and damage bacterial cells with the red fluorescent dye, Propidium Iodide (PI, 1 μL/mL); 63X magnification, scale bar: 25 μm. (**b**) Quantification of alive and damaged biomass in the function of the TL concentration. The alive and dead cells ratio was measured as 0.70 in the control, and 0.74 in the TL treated sample—confirming the antibiofilm activity of the peptide.

Finally, we checked the effect of the peptide on the live/dead cell ratio when TL was incubated in the presence of preformed biofilm. Figure 6a shows the CLSM images of treated (iii,iv) and untreated biofilms (i,ii), stained with SYTO^®^ 9 (i,ii), and PI (iii,iv) in green and red channels, respectively. The corresponding quantitative estimation of both alive and dead bacterial cells is shown in Figure 6b. As expected, the live/dead cell ratio remained unchanged following treatment with a subMIC concentration of TL (100 μM), indicating that the peptide exerted an effective antibiofilm action without increasing the number of damaged cells. These results were very similar to those obtained when TL was incubated with the bacterial suspension at the inoculation process, confirming the antibiofilm properties of TL.

## 3. Discussion

The biofilm matrix plays an important role in developing antibiotic resistance, providing a safety barrier for bacteria against the host immune system, hostile environmental conditions, and penetration of antimicrobial agents [3]. Biofilms are ubiquitous and can grow on either biotic or abiotic surfaces, including medical devices, becoming dangerous for human health, and their treatment, today, is mostly ineffective. Recently, multidrug-resistant bacteria have impelled microbiological studies towards AntiMicrobial Peptides from several organisms as new antimicrobial agents, against human infections [24]. Actually, some AMPs have shown antibiotic and antibiofilm activity on several pathogenic microorganisms, including viruses, parasites, and bacteria. For these characteristics, they have been considered effective alternatives to conventional antibiotics as they are less inclined to trigger resistance [18]. AMPs have been reported to interact with bacterial membranes, due to their hydrophilic and hydrophobic domains and to their positive net charge. However, some AMPs also showed intracellular targets, and recently, we have demonstrated that TL can act on *E. coli* FtsZ protein to impair cell division [23].

The activity of AMPs on biofilm formation have widely been studied, even if the experiments were usually performed in stagnant fluid conditions, usually very far from reality. In this work, we investigated the antibiofilm effect of TL on *Pseudomonas fluorescens* biofilms in both static and dynamic conditions. Biofilms were formed using a commercial microfluidic chamber, able to simulate the real flow conditions occurring in blood vessels or catheters lumens in very throughout-manner. Experiments were carried out using TL at different sub MIC concentrations and using a single value of volumetric flow rate (0.1 μL/min), which corresponds to a shear rate value of 1 s^−1^. The established flow conditions were in the laminar regime.

Quantification of the biofilm morphology by confocal microscopy and image analysis techniques showed that TL exhibited effective antibiofilm properties on *P. fluorescens* biofilms. Preliminary tests were carried out in static conditions to evaluate the effect of TL treatment on biofilm growth. Crystal Violet assays and confocal microscopy analyses demonstrated a strong antibiofilm activity at the highest examined TL concentration, since the biofilm architecture was strongly altered upon peptide treatment. Indeed, from the image analysis of z-stacks, biofilms appeared less dense and thinner in the presence of the antimicrobial peptide. Perhaps impairment of biofilm formation by TL might lead to detachment of bacteria cells from substrates. This effect is similar to that reported for Temporin B (TB) that was shown to inhibit biofilm formation on *Staphyloccocus epidermidis* [25]. In this paper, the authors argued an inhibitory effect of TB associated with cell wall permeabilization. Moreover, TB showed antimicrobial activity against bacteria into preformed *S. epidermidis* biofilms, confirming its potential to perform on metabolically quiescent cells. Other AMPs have already been demonstrated to affect biofilm formation. Besides its key role in modulating the innate immune response, the LL-37 peptide, was shown to prevent the formation of *P. aeruginosa* biofilm at subMIC concentrations by down-regulating transcription of Las and Rhl, two quorum-sensing systems [26]. Moreover, this peptide can inhibit initial biofilm attachment by down-regulates the expression of genes associated with the assembly of flagella involved in the process of initial adherence [27].

Next, we studied the antibiofilm effect of TL under in-flow conditions. TL was first dissolved in the bacterial suspensions, and the resulting solution was used as inoculum. Biofilm morphology was clearly modified upon the TL treatment. Untreated biofilms showed the typical patchy morphology which is observed under laminar flow as already reported by several papers [11,12,28,29,30], while at increasing sub MIC peptide concentrations, biofilm structures appeared increasingly disaggregated, showing a significative reduction of cell biomass, and biofilm surface properties (i.e., thickness and roughness coefficients).

The capability of TL to affect mature biofilm was also considered by tailored in-flow experiments carried out on preformed biofilm using the highest examined sub MIC TL concentration. Following TL treatment, a large portion of the biofilm architecture was clearly perturbed with a significant decrease of all the biofilm surface properties and the overall biomass. These results indicated that TL might also exert its antibiofilm activity on mature biofilms suggesting that TL might be capable of penetrating the preformed biofilm architecture, with a still unknown mechanism affecting biofilm structure.

The effects of the antimicrobial peptide observed on *P. fluorescens* biofilm should be due to effective antibiofilm properties of TL as all the experiments were carried out with subMIC concentrations of TL. However, we further examined this question by measuring the live/dead cell ratio following TL treatment either during biofilm growth or on preformed biofilm using a double-staining method. Confocal microscopy images and morphological parameters showed a decrease of both dead and alive cells in treated biofilms in either condition with respect to the untreated control. This phenomenon might be related to the capability of the peptide to induce biofilm eradication and perhaps to the low mass transfer resistance of biofilms cultivated under the imposed flow conditions. However, the calculated live/dead cell ratio remained unchanged in the absence and in the presence of the peptide, while the biofilm architecture was disrupted. Alteration of biofilm architecture had then to be ascribed to an effective antibiofilm activity of the peptide as no increase of damaged cells was observed following TL treatment of either nascent or mature biofilms. A further investigation is however needed to elucidate the role of the flow on the antibiofilm properties of TL, since the hydrodynamic conditions are responsible for modulating the resilience of the bacterial communities and of the delivery of small molecules to and within the biofilm structures [31].

## 4. Materials and Methods

### 4.1. Temporin L

Synthetic TL was synthesized on a solid phase by Fmoc chemistry, as reported in Di Somma et al. [23]. The stock solutions of peptide were prepared in sterile water.

### 4.2. Microorganism and Culture Conditions

*Pseudomonas fluorescens* AR 11 was purchased by DSMZ (Braunschweig, Germany). Although the fact that is considered less virulent than *P. aeruginosa*, *P. fluorescens* is known to have functional traits, able to cause human infections and contamination of equipment associated with intravenous infusion [32,33].

Bacterial cells were grown in a sterile M9 minimal medium supplemented with succinic acid (4 g/L) and made of the following ingredients: NaHPO_4_ (6.8 g/L)_,_ KH_2_PO_4_ (3 g/L), NaCl (0.5 g/L), NH_4_Cl (1 g/L), MgSO_4_·7H_2_O (95 mg/mL), CaCl_2_·2H_2_O (5.85 mg/mL) and oligo-elements solution (consisting in 0.198 g/mL EDTA, 2 mg/mL FeCl_3·_6 H_2_O, 0.21 mg/mL ZnCl_2_, 0.03 mg/mL CuCl_2_·2 H_2_O, 0.025 mg/mL CoCl_2_·2H_2_O, 0.025 mg/mL H_3_BO_3_ and 0.004 mg/mL MnCl_2_·4 H_2_O) at pH 7. Bacterial cells were cultivated at 30 °C in shaking conditions (90 rpm, Minitron Infron HT, Switzerland) overnight. The obtained bacterial suspensions were diluted to have an OD_600nm_ ≈ 0.5 to be used as inoculum.

### 4.3. Bacterial Cell Growth and Susceptibility Assay

The cell strain of *P. fluorescens* was incubated overnight in the medium at 30 °C. The cultures were used to inoculate 1:100 in fresh medium and growth at 30 °C. Reached the value of 0.5 OD/mL, different concentrations of TL were added to bacteria, and growth profiles were monitoring at OD_600nm_ for 24 h with and without treatment.

The minimum inhibitory concentration (MIC) of TL was calculated by broth microdilution. The bacterial strains were incubated overnight, and the cultures were diluted approximately to 5 × 10^5^ CFU/mL, and 50 μL of the bacterial suspension was added to ten wells and incubated with serial dilutions of peptides from an initial concentration of 512 μM. The sterility control well contained 100 μL of the medium, while the growth control well contained 100 μL of microbial suspension. The plates were incubated at 30 °C for 24 h grown, and the MIC was determined by the lowest concentration showing no visible growth by measuring the absorbance at 600 nm. For the minimum bactericidal concentration (MBC), the MIC well and the wells with a concentration higher than MIC value were plated on an agar plate and incubated at 30 °C for 24 h. The MBC value was determined as the amount of TL, allowing no colony growth from the directly plated content of the wells. MIC and MBC values were determined by three independent experiments.

### 4.4. Experimental Conditions

#### 4.4.1. Static Conditions

Preliminary tests on TL were performed under stagnant conditions. TL-treated biofilms were formed over 12 aerated well-microplates at 30 °C. Each well was filled with 2 mL of sterile minimal medium and 200 μL of the bacterial suspension. TL was added, together with the bacterial suspension, in each well at the following concentrations in the resulting solution: 25 μM, and 100 μM, respectively. During the tests, negative controls (i.e., without TL) were also formed inside the multiwell microplates. Experiments were carried out for 72 h, and each experimental condition was done in triplicate.

To assess the antibiofilm properties of TL, the Crystal Violet assay (Merck & Co. Kenilworth, NJ, US) was selected. Biomass attached to the wall of the multiwell microplates was evaluated according to the protocol of Christensen et al. [34]. Microplates were stained with 0.1% (*w*/*v*) Crystal Violet solution and incubated for 10 min. Rinsing with filtered bi-distilled water was carried out to remove the excess of Crystal Violet. Next, Crystal Violet was solubilized in 50% ethanol/water solution for 10 min. Samples optical density was measured at 590 nm (OD_590nm_).

Biofilms were also cultivated on glass substrates (18 mm × 18 mm) under stagnant fluid conditions. With the goal to investigate biofilm architecture in the presence of TL at the concentrations reported above. Briefly, glass substrates, minimal medium solution, and the bacterial suspension together with TL at the different concentrations, were inserted in each well, TL-treated biofilms were formed on both sides of the glass substrates and around the multiwell walls. Experiments were carried out for 72 h. Multiwell plates consisting of minimal medium solution, bacterial suspension, and glass substrates, were selected as a negative control of the experiment. Subsequently, the antibiofilm properties of TL on biofilm formed on glass substrates under static conditions, were examined via Confocal Laser Scanning Microscopy (CLSM), according to the procedure reported in Section 4.5.

The minimum biofilm eradication concentration (MBEC) of TL was determined. The bacterial cell was inoculated in wells and incubated at 30 °C without shaking, to allow bacterial attachment. Non-adherent cells were removed, and the well were washed three times with sterile PBS. Serial twofold dilutions of TL (initial concentration 512 μM) were added to the microplates followed by incubation at 30 °C for 24 h. After the incubation, the broth was removed, and wells were washed three times with sterile PBS, and the sterile medium was added, followed by incubation for 24 h. MBEC was defined as the minimal concentration required for eradicating the biofilm. All determinations were performed in triplicate.

#### 4.4.2. In-Flow Experimental Set-Up

##### Description of the Experimental Procedure

**Figure 7:**
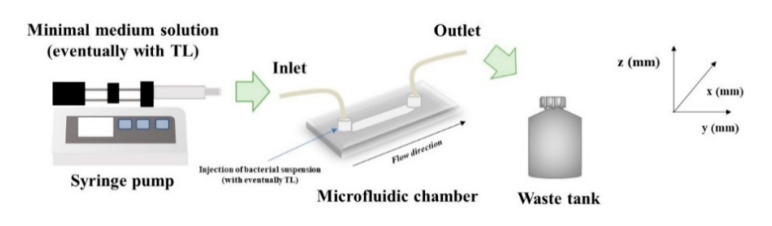
The schematization of the in-flow experimental apparatus.

The antimicrobial properties of TL were then investigated for biofilms cultivated under in-flow conditions, following an already validated approach [35]. Briefly, a continuous experimental set up made of a commercial microfluidic chamber (17 mm × 1 mm × 0.1 mm, Ibidi Cell in Focus, μ-Slide VI 0.1, Ibidi GmbH) and other auxiliary units, was selected. A schematization of the experimental apparatus is shown in Figure 7. The microfluidic chamber had six channels, the internal volume of each channel was 1.7 μL, and the thickness, i.e., channel size along the observation direction, was 100 μm.

From a feeding reservoir, the minimal medium solution was conveyed through a glass syringe pump, made of two 10 mL syringes (Harvard Apparatus, Pump 11, Pico Plus Elite, Hollston, MA, USA), to the microchamber using 1.6 mm silicone tubes. Next, a waste tank was used to collect the downstream flow. Before use, the in-flow apparatus was sterilized using a 6% sodium hypochlorite solution and then rinsed with sterile bi-distilled water. After the initial preconditioning with a fresh nutrient medium, 1 mL bacterial suspension (OD_600nm_~0.5) was injected directly to the channels, and the liquid flow was shut down for 2 h to promote bacterial cells settle. Then, experiments were carried out at a constant flow rate, Q, equal to 0.1 μL/min for 72 h. The growth temperature was kept constant at 30 °C in an. incubator.

Two different experimental campaigns were carried out to assess the antibiofilm properties of TL on biofilms developed under well-controlled flow conditions at different incubation times. In the initial experimental campaign, TL was dissolved in the bacterial suspension, and the resulting solution (inoculum + peptide) was injected into the microfluidic chamber. After 2 h in which the flow was stagnant, the experiments were carried out at a constant flow rate. Therefore, the antimicrobial properties of TL were investigated during the bacterial attachment phase. Briefly, two different concentrations of the peptide in the bacterial suspension were tested: 25 μM and 100 μM. Each in-flow test was carried out also without TL to be used as a negative control of the experiment.

In the second experimental campaign, TL antibiofilm properties were investigated on 72 h-old formed biofilms, and therefore, on mature systems. More specifically, TL was dissolved in the minimal medium solution, and the obtained solution (minimal medium+ peptide) was conveyed at a constant flow rate, to the microfluidic chamber, hosting the 72 h old biofilms, for the next 24 h.

In this case, only the highest examined concentration of TL (100 μM) was selected. In addition, experiments were also carried out without TL to be used as negative controls.

#### 4.4.3. Flow Characterization

The shear rate at the wall, $\dot{\gamma_{w}}$(s^−1^) of the microfluidic chamber was evaluated according to Equation (1), by assuming the laminarity of the flow regime according to the correlation reported by Bird et al. [36].

(1) $${\dot{\gamma }}_{w}=\frac{3\;Q}{\left(2\;\delta^{2}\cdot W\right)}$$

where ***δ*** and W are the half thickness and the width, respectively, of the rectangular section of the microfluidic chamber, in our case ***δ*** = 50 μm and ***W*** = 1 mm. For the examined flow condition (***Q*** = 0.1 μL/min), $\dot{\gamma_{w}}$ was equal to 1 s^−1^. Furthermore, the wall shear stress, $\tau_{w}$, is evaluated according to Equation (2).

(2) $$\tau_{w}={\dot{\gamma }}_{w}\cdot \eta =\frac{3\;Q}{\left(2\;\delta^{2}\cdot W\right)}\cdot \eta$$

where η (Pa·s) is the medium viscosity (also considered approximately equal to the one of water at 25 °C). Stress value ($\tau_{w}\;≅$ 0.01 Pa) corresponds to the hydrodynamic conditions typically established in capillaries and venules, and catheter lumens, according to the estimate reported by Weaver et al. [5].

For the selected hydrodynamic condition, Reynolds number, ***Re***, was evaluated as it follows:

(3) $$Re=\frac{\rho \cdot v\cdot D_{h}}{\eta }$$

where ρ (kg m^−3^) is the medium density (considered approximately equal to the one of water at 25 °C), v is the average velocity (m/s) within the channel, $D_{h}$ is the channel hydraulic diameter (m). The hydraulic diameter can be evaluated according Equation (4), and it is equal to the wetted area over the wetted perimeter.

(4) $$D_{h}=\frac{4A}{P}=\frac{4\;\left(W\cdot \delta \right)}{W+2\delta }$$

For the selected operative condition, Reynolds number of the order of 10^−3^, corresponding to the laminar regime, typical of the microfluidic conditions. Detailed analysis of the role of flow intensity on transport phenomena in biofilm growth was discussed in Recupido et al. [35].

### 4.5. Confocal Microscopy and Image Analysis

Antibiofilm properties of TL were investigated by a quantitative analysis of biofilm morphology, via Confocal Laser Scanning Microscopy (CLSM). After 72 h, samples cultivated in both stagnant and in-flow conditions were rinsed with sterile bi-distilled water to remove planktonic cells. The sample were then double-stained. Different samples were obtained to assess the antibacterial/antibiofilm properties of TL. First, biofilm morphology upon the TL treatment was investigated. TRITC conjugate ConA (Tetra-methyl-rhodamine conjugated Concanavalin A, Molecular Probes, Invitrogen, at 100 μg/mL in sterile bi-distilled water, was used to stain α-mannose residues within biofilm matrix in the red channel, while the green fluorescent dye, SYTO^®^ 9 (from LIVE/DEAD BacLight Bacterial Viability Kit, Molecular Probes, Invitrogen, Carlsbad, CA, USA) at the concentration of 3 μL/mL in filter bi-distilled water, was selected to stain the alive bacterial cells. Bacterial viability was examined too. To do so, alive cells were stained with the green fluorescent dye, SYTO^®^ 9 as reported above. Red fluorescent dye Propidium Iodide (PI), was added at the concentration of 1 μL/mL to stain damaged cells. In each case, sample staining was performed using 200 μL of each dye and by incubating samples in the darkness for 30 min at room temperature. The samples were rinsed with bi-distilled water to remove the excess of dyes.

Images were acquired using an inverted fluorescent microscope (Leica TCS SP8 STED 3x, Wetzlar, Germany). Images were obtained using HCX PL APO CS 63X/1.40 oil immersion objective and Hybrid detectors. Z-stacks of biofilm images were obtained along with the thickness of the samples and acquired using 1 μm intervals at random positions of the samples. Stack images of XY area of 10^4^ μm^2^ were obtained and recorded as tiff files and processed by Leica Las X software. A quantification of biofilm morphology was performed using image analysis techniques. More specifically, z-stacks were quantified using COMSTAT 2.1, a MATLAB© script implemented by Heydorn et al. [37], and then developed in the free-software Image J by Vorregaard [38]. Analysis of the green channel (Live/Dead staining) provided an estimate of alive cell biomass (μm^3^/μm^2^). Cell biomass (μm^3^/μm^2^) was calculated as the number of biomass pixels in all images of a stack multiplied by the voxel size, divided by the substratum area of the image stack. Analogous analysis on the red channel (obtained by Propidium Iodide staining) of the same stacks of images provided a measure of the biomass of damaged bacteria.

Surface parameters, such as the surface covered by the biofilm over the total acquired area (%), maximum thickness (μm), and dimensionless roughness coefficient, ***Ra*** (-) were also obtained by processing red-channel-stack-images from TRITC ConA staining in the red channel under different growth conditions. Ra was evaluated according to Equation (5), as reported by Heydorn et al., and Recupido et al. [35,37].

(5) $$R_{a}=\frac{1}{N}\overset{N}{\underset{i=1}{\sum }}\left|\frac{L_{i}-L_{F}}{L_{F}}\right|$$

where $L_{i}$ is the *i*-th individual thickness measurement (μm), $L_{F}$ is the mean thickness (μm) and N is the number of thickness measurements.

A quantitative comparison between TL-treated and untreated control samples was done. To guarantee the statistical significance of the analysis, three random areas of glass coupons and of the microfluidic channel, were imaged for each experiment run in stagnant or in-flow condition. Each experimental condition was repeated at least six independent times, i.e., by developing the biological samples in the same growth conditions.

## 5. Conclusions

In this work, the antibiofilm properties of TL were examined on *P. fluorescens* biofilm cultivated under two defined conditions: Static and well-controlled flow conditions using subMIC concentrations of the peptide. For in-flow experiments, a commercial microfluidic chamber was selected to tailor the hydrodynamic conditions to achieve specific values of the shear rate. In these conditions, the antibiofilm properties of TL were investigated on either nascent or mature biofilms by quantifying biofilm morphology using confocal microscopy. The viability of bacterial cells upon peptide treatment was also investigated.

The results obtained from this experimental work suggest an antibiofilm activity of TL on *P. fluorescens* biofilm is well separated from its antimicrobial properties. A number of hypotheses can be elaborated on the molecular mechanisms by which TL might interfere with biofilm formation: (i) Binding to the cell surface and inhibition of cell-cell adhesion; (ii) binding to bacteria cells determining separation from the preformed matrix; (iii) binding to bacterial surface and inhibition of bacteria interaction through maturation; (iv) binding to EPS constituents interfering with the establishment of biofilm architecture. Finally, TL might also affect the *P. fluorescens* gene expression, interfering with bacterial motility, or down-regulating genes involved in the synthesis of biofilm components. Although more investigations are needed to clarify the mechanism of biofilm impairment, these results open the way to the possibility of using immobilized TL to counteract the formation of *P. fluorescens* biofilm and to restrict its deleterious effects.

## Supplementary Materials

Supplementary Materials can be found at https://www.mdpi.com/1422-0067/21/22/8526/s1. Figure S1: Growth profiles in the presence and in the absence of different concentrations of TL.

## Abbreviations

EPS	Exopolysaccharides
AMPs	Antimicrobial peptide
TL	Temporin L
CLSM	Confocal Laser Scanning Microscopy
TRITC	Tetra-methyl-rhodamine
ConA	Concanavalin A
PI	Propidium Iodide
