# Supplementary material for: Antibiofilm Properties of Temporin-L on Pseudomonas fluorescens in Static and In-Flow Conditions
Source: Int J Mol Sci. 2020 Nov 12;21(22):8526. doi: 10.3390/ijms21228526 (PMC7696879; doi:10.3390/ijms21228526)
Supplement: Supplementary file 1 [file ijms-21-08526-s001.pdf]

Supplementary figure 1

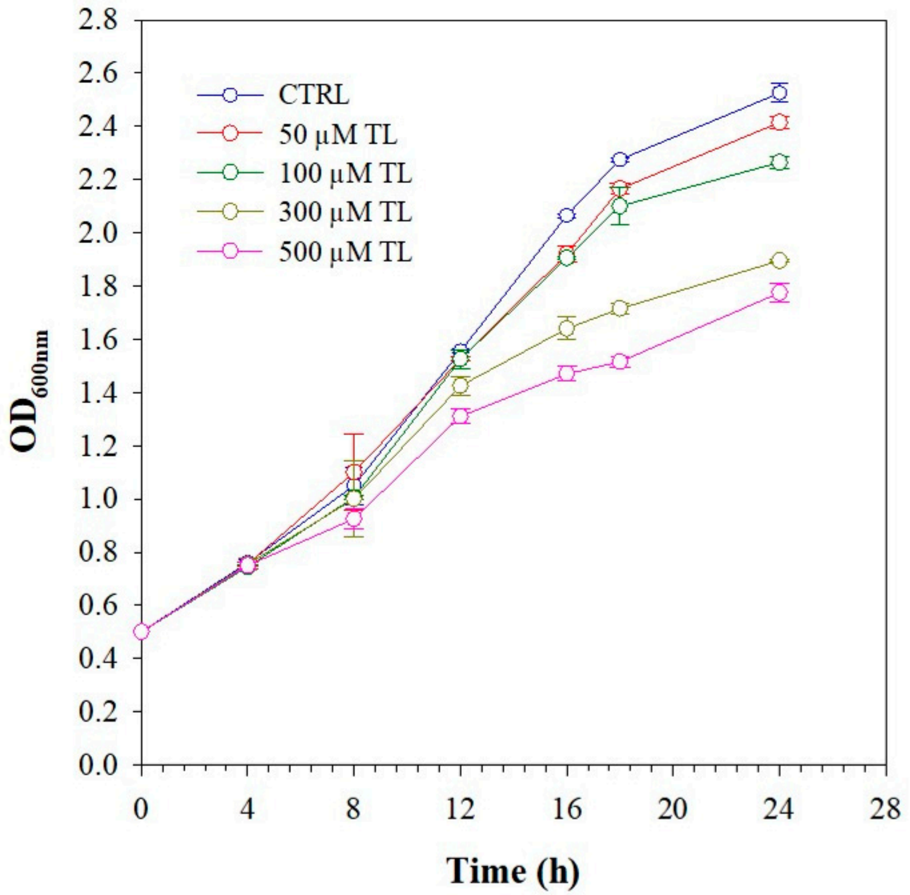

Supp. Fig 1. Growth profile in the presence and in the absence of different concentration of TL.
